# Knowledge, Attitude, and Practice Related to Malaria Diagnosis among Healthcare Workers in Hospitals: A Cross-Sectional Survey

**DOI:** 10.1155/2019/1414079

**Published:** 2019-06-11

**Authors:** Kwuntida Uthaisar Kotepui, Manas Kotepui, Chuchard Punsawad

**Affiliations:** ^1^Medical Technology Program, School of Allied Health Sciences, Walailak University, Nakhon Si Thammarat 80160, Thailand; ^2^School of Medicine, Walailak University, Nakhon Si Thammarat 80160, Thailand

## Abstract

Malaria is a potential medical emergency and should be treated immediately because delays in diagnosis and treatment are the leading causes of death in many countries. This study aimed to assess the knowledge, attitude, and practice related to malaria diagnosis for early detection among healthcare workers in the laboratories of hospitals in Thailand. The design of the study was a descriptive cross-sectional study carried out between January 2016 and March 2017 at 11 hospitals in Thailand. The interviewees included any scientists who were currently working in a medical laboratory. Mean scores for knowledge, attitude, and practice for each healthcare group were calculated and compared between groups. Data analysis was performed using the SPSS version 11.5 software package (SPSS Inc., Chicago, IL, USA). Among a total of 118 healthcare workers, most of the healthcare workers had fair to good knowledge, attitude, and practice related to malaria detection. Among the various positions of healthcare workers, medical technologists possessed a greater knowledge on malaria detection than medical technician assistants or laboratory assistants (X2 = 9.822, d.f. = 2, and P value=0.007). This study infers that knowledge, attitude, and practice related to malaria detection among healthcare workers in laboratories were adequate. However, some points of knowledge and practice must be updated. There is a very urgent need to update knowledge on malaria, especially about the number of* Plasmodium* species causing relapse in malaria patients. In addition, there is an urgent need to update the practice related to malaria detection, especially about the staining process for early detection of malaria.

## 1. Introduction

In the year 2015, there were 212 million new cases of malaria worldwide [1]. The WHO reported that the African region accounted for the most global cases of malaria at 90%, followed by the Southeast Asian region at 7% and the Eastern Mediterranean region at 2%. In addition, there was an estimate of 429,000 malaria deaths. Most of these deaths occurred in the African region (92%), followed by the Southeast Asian region (6%) and the Eastern Mediterranean region (2%) [1]. In Thailand, the predominant cause of malaria illness is infection with* Plasmodium falciparum* and* Plasmodium vivax* ratio 1:1 [1]. The ultimate goal of the regional strategy is to eliminate malaria by 2030 and to specifically eliminate* P. falciparum* malaria, considering the urgent action required because of multidrug resistance by that malaria in the Greater Mekong Subregion (GMS) countries. The principle strategy consists of the following: accelerate detection using combinations of interventions tailored to local contexts; improve detection of malaria cases and entomological surveillance, monitoring, and evaluation; and provide equity in accessing medical services [2]. In Thailand, malaria diagnosis services in the public sector are available free of charge.

Malaria detection and diagnosis involve identifying malaria parasites or antigens/products in a patient's blood. In the laboratory, the gold standard of malaria detection is using conventional microscopic diagnosis by staining thin and thick peripheral blood smears [3]. The advantage of the microscopic diagnosis technique can be attributed to its simplicity, low cost, and ability to identify the presence of parasites, including their species and density. The disadvantage is due to the staining and interpretation processes, which are labor intensive, time consuming and require well-trained healthcare workers, particularly for identifying species accurately at low parasite levels [4]. Other concentration techniques of parasite detection are also used, e.g., quantitative buffy coat (QBC) method, rapid diagnostic tests [5–10], and molecular diagnostic methods, such as polymerase chain reaction (PCR) [11]. However, these techniques require specialized instrumentation, are more costly than conventional light microscopy, and are poor at determining species and number of parasites [4].

Prompt and effective case management of uncomplicated malaria is a critical element of malaria control. These involve accurate clinical assessment, ranging from laboratory confirmation of malaria to treatment with an effective antimalarial drug [12]. Although the awareness level of malaria treatment has improved over time, some studies have shown marked variations among healthcare providers, but clinicians consistently demonstrate retention and use of knowledge and good practice [13, 14]. Thus, conducting a study on the knowledge, attitude, and practice related to malaria detection among healthcare providers may help to improve the quality of care and serve as baseline information to improve malaria diagnosis.

## 2. Materials and Methods

The study protocol was approved by the Ethical Clearance Committee on Human Rights Related to Researches Involving Human Subjects of Walailak University. The design of the study was a descriptive cross-sectional study carried out between January 2016 and March 2017 at hospitals in Thailand. The interviewees included any healthcare workers in laboratories of hospitals. This included medical technologists, medical technician assistants, and laboratory assistants. Informed consent was obtained from all respondents. Respondents were given the right to refuse to take part in the study as well as to withdraw anytime during the interview. Privacy and confidentiality were maintained throughout the study.

The sampling technique used in this study was convenient sampling. The respondents included all the healthcare workers in the hospitals who have agreed to respond. Data were collected using a structured questionnaire specifically developed for this purpose. The first part of the questionnaire included questions about the sociodemographic characteristics of the participants. The second part of the questionnaire included questions about the knowledge, attitude, and practice related to malaria diagnosis. The questionnaires were translated into the Thai language. All questions were closed-ended in structure.

Knowledge on malaria was assessed by requesting respondents to answer questions about malaria transmission, malaria species, severity of malaria, the cycles of malaria parasites, relapses, incubation period, quartan malaria, symptoms of malaria, and the gold standard method of malaria detection (Table 2). Each correct response was awarded one (1) point and each wrong response was scored zero (0). The total score ranged from 0 to 9. Respondents with scores of 0-3 were considered to have poor knowledge, those with 4-6 points were considered to have fair knowledge, and those with 7-9 points were considered to have good knowledge.

Attitude on malaria detection was assessed using questionnaire by requesting respondents to answer questions in regard to time, expertise, expense, and comprehensiveness when utilizing the blood film technique to detect malaria. Questions were also asked about other detection techniques (such as dipsticks with HRP-2 and pLDH) and their comparison with the blood film technique in regard to sensitivity and specificity (Table 3). The total score ranged from 0 to 5. Respondents with scores of 0-1 were considered to have poor attitude, those with 2-3 points were considered to have fair attitude, and those with 4-5 points were considered to have good attitude.

Practice of malaria detection was assessed by requesting respondents to answer questions about the technique in regard to Giemsa staining, the amount of blood used, fixing, white blood cell staining, the process of preparing and storing Giemsa dye, and the process of determining the parasite in microscope (Table 4). The total score ranged from 0 to 10. Respondents with scores of 0-3 were considered to have poor practice, those with 4-7 points were considered to have fair practice, and those with 8-10 points were considered to have good practice. Mean scores for knowledge, attitude, and practice for each healthcare group were calculated and compared between groups using the Kruskal-Wallis Test. Data analysis was performed using the SPSS version 11.5 software package (SPSS Inc., Chicago, IL, USA).

## 3. Results and Discussion

In Thailand, laboratory services are an integral part of clinical decision-making and contribute to diagnostic/therapeutic decisions and disease monitoring/prevention for patients [15]. The decision of malaria treatment may rely heavily on the laboratory examination of the malaria parasite. Malaria should be diagnosed and treated immediately because delays in diagnosis and treatment are the leading causes of death in many countries [16]. Early treatment with an artemisinin-based combination treatment (ACT) and with a low-dose of primaquine prevents further transmission of malaria from human to mosquito [17]. The precise diagnosis of malaria is directly related to the level of awareness in regard to knowledge, attitude, and practice related to malaria detection among healthcare workers working in the laboratories of hospitals. Hence, this study assessed the knowledge, attitudes, and practices related to malaria detection among healthcare workers, including medical technologists, medical technician assistants, and laboratory assistants.

### 3.1. Sociodemographic Data

**Figure 1:**
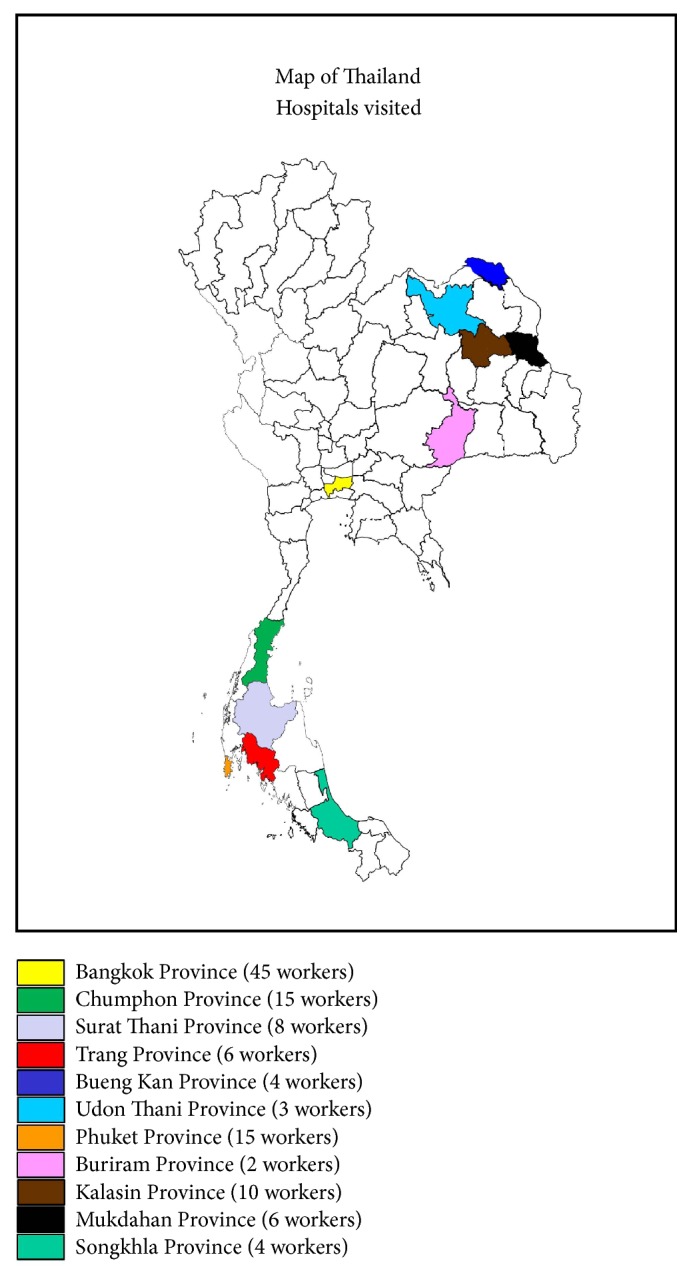
A map of hospitals enrolled in the study.

Table 1 shows the demographic data of the respondents. A total of 118 respondents at 11 hospitals in Thailand participated in the study (Figure 1). Most of the respondents were aged between 20 and 29 (60, 50.8%), followed by 30-39 (34, 28.8%), 40-49 (12, 10.2%), 50-59 (11, 9.3%), and 60 and over (1, 0.8%). The results of this study showed that most of the respondents were young and at an age between 20 and 29 years old. This indicates that young workers are employed in the medical laboratory of hospitals. This was the same age range reported in a previous study assessing laboratory practices of healthcare workers carrying out blood film microscopy for diagnosis of malaria parasites in Ethiopia [18]. This study showed that most respondents had a bachelor's degree (101, 85.6%) and an income between 21,000 and 30,000 baht per month (50, 42.4%). Ninety-six respondents (81.4%) were medical technologists, 12 (10.2%) were medical technician assistants, and 10 (8.5%) were in other positions (e.g., laboratory assistants). Most of the respondents were employed in government hospitals (106, 89.8%) with a mean working experience of about nine years. In the medical laboratory, medical technologists, medical technician assistants, and laboratory assistants were working together. The medical technologist, who has a bachelor's degree from a university, was found mostly in the medical laboratory of hospitals. The role of the medical technologist at the hospital is to be in charge of classes for medical tests, quality assurance, reading the results of medical tests, proving the test results, and then sending them to medical doctors. The medical technologist performs all laboratory disciplines: clinical chemistry, hematology, blood banking, microbiology, serology/immunology, and histotechnology. The medical technologist is always adjusting to technical advances. In a few more years, the medical community will require greater numbers of better trained medical technologists to meet these emerging challenges in health care [19]. In Thailand, the Ministry of Public Health supports training and promotes improvement of the quality of medical technology and laboratories. Professional organizations, including the Association of Medical Technology of Thailand and the Medical Technology Council, support technical training and promote networking among medical technologists [15]. In this study, the medical technician assistant, who graduated with a certificate degree in medical sciences, was the second most common enrolled. In Thailand, medical technician assistants have an opportunity to further study in a medical technology program in order to become medical technologists. This program is supported by funding from the government or hospitals. The results also showed that most of the respondents were employed in government hospitals. However, this may be due to the process of randomly selecting respondents, which happen to be mostly employed in government hospitals. Most medical technologists want to work in government hospitals because they want to become government officials. However, a previous study showed that medical technologists had low scores in their attitude in regard to pay, professional status, medical technologist-physician relationship, and job-task requirements. Therefore, this suggested a need to improve job satisfaction among these healthcare workers [20].

### 3.2. Knowledge about Malaria

The results of the knowledge assessment about malaria detection among respondents are shown in Table 2. Results revealed that 27 respondents (22.9%) had good knowledge, while 88 (74.6%) had fair knowledge and 3 (2.5%) had poor knowledge on malaria detection. For “yes” as a correct answer, a majority of the sample group knew that malaria is transmitted by anopheles mosquitos (111, 94.1%);* P. falciparum* causes severe malaria (115, 97.5%); quartan malaria is caused by* P. malariae* (94, 79.7%); symptoms of malaria infection include cold, hot, and sweating stages (96, 81.1%); and the gold standard method of malaria detection involves the detection of the malaria agent in thick or thin blood film (113, 95.8%). For “no” as a correct answer, a majority of the sample group did not know that more than four species of malaria are transmitted to humans, including* P. knowlesi* malaria (83, 80.3%); malaria cycles include the human stage and mosquito stage (102, 86.4%);* P. vivax* and* P. ovale *cause relapse in malaria patients (63, 53.4%); and incubation period means the time elapsed between exposures to the presence of symptoms (90, 76.3%). There were different trends in knowledge. Medical technologists had higher knowledge than those of medical technician assistants and other positions (X2 = 9.822, d.f. = 2, P value=0.007, and Kruskal-Wallis Test). The diagnosis of malaria in clinical laboratories mainly depends on blood smear microscopy, and this technique remains the most widely used in Thailand. Despite the importance of blood smear microscopy for patient diagnosis and treatment, little effort has been made to precisely determine sources of error in malaria smear microscopic diagnosis. In regard to the knowledge of the respondents, results revealed that most of the respondents had good to fair knowledge about malaria detection. This confirmed that all respondents who were responsible for malaria detection had adequate knowledge about malaria. However, there was a lack of knowledge among these respondents about the number of* Plasmodium* species which can infect humans. Nowadays, there are five or six species of malaria that can infect humans, including* P. falciparum, P. vivax, P. knowlesi *[21],* P. ovale curtisi*, and* P. ovale wallikeri *[22]. Most of the respondents also did not know precisely that malaria cycles include the human stage and mosquito stage, and also most of the respondents did not know that* P. vivax* and* P. ovale* cause relapse in malaria patients [23]. Moreover, most of the respondents did not know that the incubation period means the time elapsed between exposures to the presence of symptoms. These misunderstandings must be emphasized and accounted for in order to improve knowledge about malaria detection among these respondents.

### 3.3. Attitude about Malaria Detection

Assessment of attitude about malaria detection among the respondents revealed that 93 respondents (78.8%) had good attitude, while 25 (21.2%) had fair attitude about malaria detection (Table 3). A majority of the sample group believed that malaria detection by blood film technique is time consuming (47, 39.8%); malaria detection by blood film technique requires skillful expertise (111, 94.1%); malaria detection by blood film technique requires high expenses (16, 13.6%); malaria detection by blood film technique can differentiate the four species of malaria (103, 87.3%); hospitals in malaria endemic areas require other malaria detection techniques such as dipsticks, PCR, etc. (95, 80.5%); and dipsticks with HRP-2 and pLDH have higher sensitivity and specificity than the blood film technique (92, 78%). There were no different trends in attitude among medical technologists, medical technician assistants, and other positions (X2 = 1.568, d.f. = 2, P value=0.457, and Kruskal-Wallis Test). In regard to the attitude of the respondents, results revealed that most of the respondents had good to fair attitude about malaria detection. They believed that malaria detection by blood film technique is time consuming. Generally, blood film preparation needs about 30 minutes for each sample which is quite time consuming. In addition, the average time spent to read a single blood smear slide was 10.8 minutes, with the minimum being 2 minutes and the maximum being 45 minutes [18]. They also thought that malaria detection by the blood film technique was expensive, which is not true. The blood film technique requires no more than 100 baht per case.

### 3.4. Practice about Malaria Detection

The results of the practice evaluation about malaria detection are shown in Table 4. It revealed that 96 respondents (81.4%) had good practice, while 21 (11.8%) had fair practice. For “yes” as a correct answer, a majority of the sample group knew that thick blood film with a lesser amount of blood leads to misinterpreting results (99, 83.9%); pure Giemsa requires stirring before filtering and filtering before using every time (105, 89%); blood smear should be air-dried before fixing (112, 94.9%); nucleus of white blood cells should be dark blue in case of good quality of Giemsa staining (87, 73.7%); destaining requires slowly rinsing the glass slide with tap water to completely destain (105, 89%); Giemsa diluent should be neutral at pH 7.2 (112, 94.9%); pure Giemsa should be stored in a brown bottle and tightly screwed closed to protect against evaporation and oxidation reaction (105, 89%); detection of malaria by blood film technique should be done via objective lens (106, 89.8%); and thin blood smear requires well margins of spreader and suitable drop of blood (109, 92.4%). For “no” as a correct answer, a majority of the sample group did not know that thick blood smear with Giemsa does not require fixing technique with absolute methanol (94, 79.7%). There were no different trends in practice among medical technologists, medical technician assistants, and other positions (X2 = 0.836, d.f. = 2, P value=0.658, and Kruskal-Wallis Test). In regard to the practice of the respondents, results revealed that most of the respondents had good practice related to malaria detection. This confirms that all respondents responsible for malaria detection have adequate practice in regard to malaria detection. However, a majority of the respondents misunderstood that a thick blood smear with Giemsa requires fixing technique with absolute methanol. For Giemsa staining, the thick blood film stood in 5% Giemsa for 30 minutes (does not require fixing for breaking red blood cells), and then it was washed gently with tap water and air-dried [24]. In addition, delay in preparation of the blood smear may allow for the degeneration of the cellular elements of blood and may result in a pseudothrombocytopenia (falsely reduced platelet count) due to formation of platelet aggregation [25]. Only a few studies evaluated the frequency and types of mistakes in the preanalytical process. This result appears to confirm the high mistakes in the preanalytical phase, which was mentioned in recent previous studies [26]. The need for continuous training of laboratory technicians and/or local microscopists to solve these problems at each health facility could be of paramount importance to alleviate the problem. A previous study examining the malaria microscopy diagnosis of 10 laboratories on the Thai-Myanmar border suggested that a high level of input from international laboratory technicians, provision of training, and good follow-up and evaluation were required. In addition, adequate training of national technicians especially in regard to long-term sustainability is a need that should be emphasized [27].

### 3.5. Position of Healthcare Workers and Level of Knowledge, Attitude, and Practice on Malaria

The age of healthcare workers in this study was significantly different (X2 = 12.57, d.f. = 2, P value=0.002, and Kruskal-Wallis Test). The mean age of medical technologists was 30.79±8.13 years, which was lower than that of medical technician assistants (39.67±13.57 years) and other positions (40.6±10.31 years). Knowledge related to malaria detection among healthcare workers was also significantly different (X2 = 9.822, d.f. = 2, P value=0.007, and Kruskal-Wallis Test). Medical technologists had higher knowledge (5.78±1.24) than that of medical technician assistants (4.91±0.99) and other positions (4.9±0.74). However, a difference in attitude and practice related to malaria detection was not found (P value>0.05). The results also showed that the experience of healthcare workers was significantly different among three groups (X2 = 13.477, d.f. = 2, P value=0.001, and Kruskal-Wallis Test). Medical technologists had less experience (7.29±7.17 years) than that of medical technician assistants (14.45±11.84 years) and other positions (19.44±11.54 years). The position of healthcare workers in medical laboratories was associated with their level of knowledge, attitude, and practice in regard to malaria detection. A significant difference in the age of healthcare workers in medical laboratories was found. The age of medical technologists was lower than that of medical technician assistants and laboratory assistants. This may be due to the fact that most of the medical technician assistants and laboratory assistants have been working for a longer period of time in medical laboratories when compared with the medical technologists. A significant difference in knowledge on malaria among healthcare workers was also found. Medical technologists had higher knowledge than that of medical technician assistants and laboratory assistants. Medical technologists were qualified medical laboratory technicians who received their bachelor's degree after four years of study in recognized training institutions in the country, whereas medical technician assistants received their certification after two years of study. Medical technology students must realize that they will be required to perform their duties in the clinical laboratories and interact with other healthcare professionals. They must be competent, knowledgeable, and reliable [28–30]. In addition, most of the medical technologists in this study had just graduated from their university. As for medical technician assistants, their long routine of work may have resulted in a lack of knowledge which requires regular review.

### 3.6. Government Hospitals and Private Hospitals

When comparing government and private hospitals, knowledge about malaria detection among healthcare workers in government hospitals (5.53±1.20) was lower than those in private hospitals (6.41±1.16) (X2 = 5.514, d.f. = 1, P value=0.019, and Kruskal-Wallis Test). However, differences in age, attitude, practice, and experience of healthcare workers between government and private hospitals were not found (P value>0.05, Kruskal-Wallis Test). The difference in knowledge of healthcare workers in government and private hospitals may be due to the employment of new staff at private hospitals; these new staff would have just graduated from university and frequently seek out employment in private hospitals because of the higher income offered. New staff may have fresher and more up-to-date knowledge than those who have worked long term. However, there were a lower number of respondents retrieved from private hospitals than from government hospitals for this study, which needs to be carefully interpreted. In addition, some healthcare workers leave their profession in private hospitals and look for work in government hospitals because government hospitals provide a stable career although the income is lower than that of private hospitals. Reports from the United States of America showed that 4% of medical laboratory technologists leave the profession each year because of the lack of career advancement, noncompetitive salaries, and job-related stress [31–33]. In Thailand, the Medical Technology Council, a legal organization, also monitors medical technology performance and promotes a national system for laboratory accreditation [15].

This study had limitations. First, the selection bias of convenient sampling was the main limitation of this study. Second, the assessment of practice related to malaria detection was based on the author's questionnaire, and so the actual performance and results of the staining technique at hospitals could not be observed. However, these results may represent the knowledge, attitude, and practice related to malaria diagnosis among healthcare workers at hospitals in Thailand. It is recommended that further studies should consider increasing the number of hospitals included in their study. This will strengthen and improve the information on knowledge, attitude, and practice on malaria detection throughout Thailand.

## 4. Conclusion

This study can conclude that knowledge, attitude, and practice related to malaria detection among healthcare workers in the laboratories of hospitals were found to be adequate. However, there was a need to update the knowledge of healthcare workers, especially about the number of* Plasmodium* species causing relapse in malaria patients. There was also a need to update the practice related to malaria detection, especially about the staining process in thick and thin blood smear examinations. This is because knowledge and personnel laboratory skills are fundamental for the implementation of a quality management system for the laboratory. In addition, training sessions were offered by the Medical Technology Council, the private sector, and other stakeholders.

## Figures and Tables

**Table 1:** Sociodemographic data.

Characteristics	Frequency (n=217)	Percentage
*Age (years)*		
Mean± SD	32.5 ± 9.62	
Min-Max	22-60	
20-29	60	50.8
30-39	34	28.8
40-49	12	10.2
50-59	11	9.3
>60	1	0.8
*Gender*		
Male	35	29.7
Female	83	70.3
*Marital status*		
Single	82	69.5
Married	33	28
Separated	2	1.7
Others	1	0.8
*Religion*		
Buddhist	110	93.2
Christian	1	0.8
Islam	6	5.1
Others	1	0.8
*Education*		
Secondary school	2	1.7
Certificate	9	7.6
Bachelor's degree	101	85.6
Master's degree	6	5.1
*Income (baht)*		
<5,000	2	1.7
5,000-10,000	3	2.5
10,001-20,000	39	33.1
20,001-30,000	50	42.4
>30,000	24	20.3
*Position*		
Medical technologist	96	81.4
Medical technician assistant	12	10.2
Other	10	8.5
*Experience (years, mean ± SD) *		8.97±8.87
*Type of hospital*		
Government	106	89.8
Private	12	10.2

**Table 2:** Knowledge on malaria.

Parameters	Medical technologist *∗*Freq. (of yes) %	Laboratory scientist *∗*Freq. (of yes) %	Others *∗*Freq. (of yes)%	Total *∗*Freq. (of yes)%
Is malaria transmitted by anopheles mosquito?	91 (94.8)	12 (100)	8 (80)	111 (94.1)
Are there four species of malaria transmitted to humans?	65 (67.7)	10 (83.3)	8 (8)	83 (70.3)
Do *Plasmodium falciparum* cause severe malaria?	94 (97.9)	11 (91.7)	10 (100)	115 (97.5)
Do malaria cycles include liver stage and red blood cells stage?	81 (84.4)	12 (100)	9 (90)	102 (86.4)
Do *Plasmodium falciparum* and *Plasmodium vivax* cause relapse in malaria patients?	47 (49)	8 (66.7)	8 (80)	63 (53.4)
Does the incubation period mean the time elapsed between exposure and the discovery of malaria in the blood?	70 (72.9)	10 (83.3)	10 (100)	90 (76.3)
Is quartan malaria caused by *Plasmodium malariae*?	78 (81.3)	8 (66.7)	8 (80)	94 (79.7)
Do symptoms of malaria infection include cold, hot, and sweating stages?	79 (82.3)	8 (66.7)	9 (90)	96 (81.1)
Does the gold standard method of malaria detection involve the detection of the malaria agent in thick or thin blood film?	92 (95.8)	12 (100)	9 (90)	113 (95.8)
Mean score (X±SD)	5.78±1.24	4.91±0.99	4.9±0.74	5.62±1.23

*∗*Freq. = frequency.

**Table 3:** Attitude on malaria detection.

Parameters	Medical technologist *∗*Freq. (of yes) %	Laboratory scientist *∗*Freq. (of yes) %	Others *∗*Freq. (of yes) %	Total *∗*Freq. (of yes) %
Does malaria detection by blood film technique require time?	34 (35.4)	4 (33.3)	9 (90)	47 (39.8)
Does malaria detection by blood film technique require skillful expertise?	90 (93.8)	11 (91.7)	10 (100)	111 (94.1)
Does malaria detection by blood film technique require high expenses?	11 (11.5)	2 (16.7)	3 (30)	16 (13.6)
Does malaria detection by blood film technique differentiate the four species of malaria?	83 (86.5)	12 (100)	8 (80)	103 (87.3)
Do hospitals in malaria endemic areas require other malaria detection techniques such as dipsticks, PCR, etc.?	78 (81.3)	9 (75)	8 (80)	95 (80.5)
Do dipsticks with HRP-2 and pLDH have higher sensitivity and specificity than the blood film technique?	74 (77.1)	10 (83.3)	8 (80)	92 (78)
Mean score (X±SD)	4.08±0.79	4±0.85	4.4±0.84	4.10±0.8

**Table 4:** Practice of malaria detection.

Parameters	Medical technologist *∗*Freq.%	Laboratory scientist *∗*Freq.%	Others *∗*Freq.%	Total *∗*Freq.%
Does thick blood smear with Giemsa require fixing technique with absolute methanol?	75 (78.1)	11 (91.7)	8 (80)	94 (79.7)
Does thick blood film with a lesser amount of blood lead to misinterpreting results?	80 (83.3)	11 (91.7)	8 (80)	99 (83.9)
Should blood smear be air-dried before fixing?	92 (95.8)	11 (91.7)	9 (90)	112 (94.9)
Should the nucleus of white blood cells be dark blue in case of good quality of Giemsa staining?	71 (74)	8 (66.7)	8 (80)	87 (73.7)
Do pure Giemsa require stirring before filtering and filtering before using every time?	86 (89.6)	9 (75)	10 (100)	105 (89)
Does destaining require slowly rinsing the glass slide with tap water to completely destain?	85 (88.5)	11 (91.7)	9 (90)	105 (89)
Should the Giemsa diluent be neutral at pH 7.2?	91 (94.8)	11 (91.7)	10 (100)	112 (94.9)
Should pure Giemsa be stored in a brown bottle and tightly screwed closed to protect against evaporation and oxidation reaction?	84 (87.5)	10 (83.3)	10 (100)	105 (89)
Should detection of malaria by blood film technique be done via objective lens?	86 (89.6)	10 (83.3)	10 (100)	106 (89.8)
Does thin blood smear require well margins of spreader and suitable drop of blood?	91 (94.8)	9 (75)	9 (90)	109 (92.4)
Mean score (X±SD)	8.35±1.06	8.17±1.59	8.5±1.35	8.34±1.14

## Data Availability

The data used to support the findings of this study are available from the corresponding author upon request.
